# The Evolution of Molecular Clocks: Concepts, Calibrations, and Challenges

**DOI:** 10.3390/biology15151329

**Published:** 2026-08-06

**Authors:** Pedro Soares, Teresa Rito

**Affiliations:** 1 CBMA (Centre of Molecular and Environmental Biology), Department of Biology, University of Minho, Campus de Gualtar, 4710-057 Braga, Portugal; teresarito@bio.uminho.pt; 2 Institute of Science and Innovation for Bio-Sustainability, University of Minho, Campus de Gualtar, 4710-057 Braga, Portugal

**Keywords:** molecular clock, divergence dating, substitution rate, calibration, relaxed clock, Bayesian phylogenetics, ancient DNA, time-dependent rates, phylogeography, evolutionary timescales

## Abstract

Molecular clocks use the accumulation of genetic changes to estimate the timing of evolutionary and demographic events. Since their formulation in the 1960s, molecular clocks have evolved from relatively simple strict-rate assumptions into a diverse set of statistical frameworks that include strict, relaxed, local and time-dependent models. In this review, we examine the conceptual basis of molecular-clock inference and the biological, demographic and methodological factors that influence clock estimates. We distinguish mutation rates from substitution rates and discuss how molecular, genomic, life-history, environmental and population-level processes can affect estimated evolutionary rates. We then review the main analytical approaches used to estimate branch lengths and divergence times, including maximum likelihood, Bayesian inference, parsimony-based methods and regression-based techniques; we also focus on the statistical evaluation of clock-like behaviour. Particular attention is given to calibration, as molecular clocks only become informative for absolute time estimation when genetic distances are anchored to an external temporal framework. We review calibration strategies based on fossils, geological and biogeographic events, founder events, ancient sequences, pedigrees, direct mutation-rate estimates, longitudinal sampling, secondary calibrations and externally estimated substitution rates. Finally, we discuss cases in which molecular-clock estimates show concordance or discordance with independent archaeological, palaeontological or epidemiological evidence, as well as cases where apparent agreement may reflect circular reasoning. We argue that molecular clocks remain essential tools for evolutionary biology, but that their reliability depends on explicit model testing, transparent calibration choices and careful interpretation of the results.

## Simple Summary

Scientists often want to know when species, populations, viruses, bacteria or genetic lineages separated from one another. Molecular clocks provide one way to answer this question by using the accumulation of changes in genetic material as a measure of time. However, these clocks are not simple stopwatches. Their speed can vary among organisms, genes, populations and time periods, and the dates they produce depend strongly on how they are calibrated. This review explains how molecular clocks work, why they can give different answers, and how researchers can test whether a clock is suitable for a particular dataset. We discuss different clock models, sources of biological and methodological uncertainty, and the main ways in which clocks can be anchored to real time, including fossils, geological events, ancient samples, pedigrees and directly measured mutation rates. Understanding these issues is important because molecular clocks are widely used to reconstruct evolution, human prehistory, pathogen emergence, species diversification and past environmental change. Used carefully, they are powerful tools; however, when used uncritically, they can create misleading historical narratives.

## 1. Introduction

The concept of the molecular clock was introduced in the early 1960s by Emile Zuckerkandl and Linus Pauling, who proposed that the number of amino acid differences in certain proteins between species could be used to estimate the time since their divergence [1,2]. Motoo Kimura later provided the mathematical foundation for these observations with his Neutral Theory of Molecular Evolution, which posits that most genetic variation is neutral, allowing for a nearly constant rate of molecular change: a key assumption underlying the molecular clock [3]. With advances in DNA sequencing during the 1970s and 1980s, molecular clocks began to be widely applied to DNA sequence data.

A molecular clock uses the accumulation of molecular substitutions along evolutionary lineages to estimate divergence times. However, this requires the estimation and calibration of substitution rates. Furthermore, the concept of molecular clock has evolved over time, with recognition of its limitations, the introduction of more sophisticated models and rigorous statistical validation. In this review, we outline the fundamental principles of the molecular clock, describe the factors influencing evolutionary rate estimates, discuss alternative clock models, survey key applications of the molecular clock, and provide a detailed focus on calibration methodologies. Although examples are drawn from diverse taxonomic groups, human mitochondrial DNA is used as an extended case study because its long history of molecular-clock application allows calibration, time dependency, phylogeographic concordance and circular reasoning to be examined within a single well-characterised framework.

Beyond summarizing molecular-clock models and calibration strategies, this review emphasizes two issues often treated separately: the timescale dependence of estimated evolutionary rates and the risk of circular reasoning when historical events are used for both calibration and validation. We integrate model testing, calibration choice and independent external evidence into a practical framework for evaluating molecular dates.

## 2. Factors Influencing the Molecular Clock

Several factors can influence molecular clocks, but before reviewing them it is important to distinguish several related terms. We use mutation rate for the rate at which new sequence variants arise through replication errors, DNA damage, or repair, generally expressed per nucleotide per replication, generation, or unit of time. In unicellular organisms, newly arising mutations may be transmitted directly to descendant cells, whereas in sexually reproducing species only germline mutations are heritable. We use substitution rate for the rate at which sequence replacements accumulate along evolutionary lineages, usually expressed as substitutions per site per unit of time. In classical population-genetic terms, a substitution is completed when a mutation becomes fixed in a population [4]. Because only a fraction of newly arising variants increase in frequency and eventually become fixed, the substitution rate reflects the long-term outcome of mutation, drift, and selection and underlies the traditional molecular-clock concept proposed by Zuckerkandl and Pauling [1,2]. Under a simple neutral model, the neutral substitution rate is expected to approximate the neutral mutation rate when both are expressed in equivalent temporal units [4]. Finally, we use estimated molecular evolutionary rate, or simply evolutionary rate, as a broader operational term for a rate inferred from observed molecular differences over a specified timescale. Depending on the data and temporal depth, this estimate may include recently arisen or segregating variants as well as substitutions that have persisted over longer evolutionary periods and should therefore not automatically be equated with either a directly measured mutation rate or a long-term substitution rate.

The instantaneous mutation rate is a key determinant of the substitution rate; regions or species with higher mutation rates are likely to have higher substitution rates because more genetic variants are available for potential fixation. Consequently, molecular clock estimates are often specific to particular taxonomic groups, genomes, or specific genomic regions, reflecting their unique mutational processes and evolutionary histories, including selection and demographies. There is no universal molecular clock [4,5] and, in fact, not even a single molecular clock exists for an organism [6].

**Figure 1:**
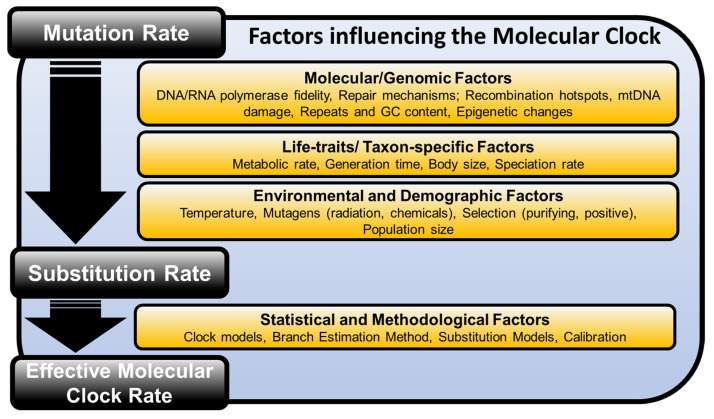
Schematic representation of the factors influencing molecular-clock rates and their estimation. The diagram distinguishes the mutation rate from the substitution rate, which traditionally underlies the molecular clock [1,2]. Four broad classes of factors are represented: molecular and genomic factors; life-history and taxon-specific factors; environmental and demographic factors; and statistical and methodological factors. The first three can influence biological mutation or substitution rates, whereas statistical and methodological factors affect the rates estimated from molecular data.

For clarity, the factors influencing the molecular clock can be grouped into four categories: Molecular/Genomic factors, which mostly influence the mutation rate; Life-history/Taxon-specific factors, which influence both mutation and substitution rates; Environmental and Demographic factors, which mainly affect the substitution rate; and Statistical and Methodological factors, which do not affect biological rates but influence the estimated molecular evolutionary rate (Figure 1). These influences are addressed in the following subsections.

### 2.1. Molecular/Genomic Factors

Under the neutral theory of molecular evolution, the long-term substitution rate of selectively neutral mutations is expected to approximate the neutral mutation rate, because the number of new neutral mutations entering a population and their probability of fixation balance each other [3]. A higher mutation rate produces more genetic variants that may eventually become fixed in a population, thereby increasing the substitution rate.

Mutations arise naturally through errors made by DNA polymerases, or by RNA polymerases in some viruses. As mentioned before, only mutations in the germline in sexually reproducing organisms can become part of the gene pool. Different DNA polymerases likely have different mutation rates. Although most have proofreading activity [7], some errors escape correction. The main replicative DNA polymerases in bacteria and multicellular organisms typically have error rates in the range of 10^−8^–10^−10^ errors per nucleotide per replication, although these rates vary among species, polymerases and genomic regions [7]. For example, DNA polymerase γ, which is responsible for replication of mitochondrial DNA (mtDNA) in eukaryotes, is associated with the high spontaneous mutation rate of mitochondrial genomes. However, this elevated rate is probably not explained solely by lower polymerase fidelity, but also by oxidative damage within mitochondria [8].

Repair mechanisms are able to decrease overall mutation rates, but repair synthesis can be less accurate than normal replication in some regions of the genome [9]. Human genomic studies also suggested that hotspots of recombination or regions of higher recombination have higher diversity [10] likely caused by the lower fidelity of repair synthesis following homologous recombination.

Structural features of the genome may also influence mutation rates. Short repetitive regions are prone to mutation through replication slippage. Some regions, including GC-rich regions, may form secondary structures [11], and methylated cytosines are more prone to deamination [12], for example.

Understanding the factors that influence mutation rates is not always required for applying a molecular clock, but it is important for interpreting mutational patterns and selecting appropriate substitution models.

### 2.2. Life-Traits, Taxon-Specific Factors

Mutation and substitution rates are specific to species, lineages and genomic regions, because many of the biological factors that shape molecular evolution are taxon-specific. These factors may alter the instantaneous mutation rate, but they may also affect hereditary dynamics, reproductive patterns and life-history traits that determine how mutations are transmitted across generations and eventually become fixed. Generation time, metabolic rate, body size, reproductive strategy and, in some cases, speciation dynamics can all influence substitution rates, but their relative importance differs substantially among taxonomic groups.

One well-studied characteristic of a species that has been associated with substitution rates is the metabolic rate of the species. The Metabolic Rate Hypothesis proposes that species with higher metabolic rates tend to have higher substitution rates [13], potentially because increased metabolic activity can elevate the mutation rate. One hypothesis is that higher metabolic rates will likely generate more reactive oxygen species, which can result in higher mutation rates [13,14]. This effect has been discussed particularly in relation to mtDNA, which is widely used in evolutionary studies. A complementary hypothesis is that higher metabolic rates may increase body temperature, which could reduce DNA polymerase fidelity and thereby increase mutation rates [15].

Another life-history trait that can alter the molecular clock is the longevity of the individual or more specifically the generation time [16]. Species with longer lifespans usually have longer generation times, resulting in fewer generations and therefore fewer opportunities for mutations to be transmitted over a specific period of time. This pattern has been observed in animals with contrasting generation times [17], including hominoids [18].

There is substantial evidence that smaller-bodied species tend to have faster estimated molecular evolutionary rates compared to larger species, especially in vertebrates [13]. Other studies suggest that such a trend cannot be extended outside the vertebrates [5]. However, even the correlation in vertebrates might not be a direct parameter, as larger animals tend to have longer generation times and lower metabolic rates [19], both contributing to slower estimated molecular evolutionary rates.

A final, more debated factor is the possible association between molecular evolutionary rates and speciation rates. Higher speciation rates may be associated with accelerated molecular evolution, especially if lineage splitting is accompanied by repeated founder events, population subdivision, ecological shifts or changes in selection. However, this relationship is less direct and more debated than the effects of generation time, metabolic rate or body size. Correlations between speciation rates and molecular clocks have been observed in plants, fishes, mammals and other taxa [20,21,22,23]. However, other authors failed to detect such a trend or detected negative correlations [24,25]. Nevertheless, even when correlations are detected between rate of speciation and substitution rate, they tend to be weaker than when comparing substitution rates with temperature, body size or generation time.

### 2.3. Environmental and Demographic Factors

Independently of species-specific mutation rates and life-history traits, substitution rates can also be influenced by the environmental and demographic conditions experienced by populations. These effects may arise through changes in the instantaneous mutation rate, through differences in the strength of both positive and purifying selection, or through variation in the probability that mutations become fixed.

Environmental temperature is one potential factor that can modify mutation rates within species. This is particularly relevant for microbes, where growth temperature can influence mutation rates [26,27]. Among animals, this could be particularly true for ectotherms. This was shown for lizards [28], including Australian Eugongylinae skinks where temperature was suggested to explain up to 45% of the observed variation in synonymous mutation rates [29]. Not surprisingly, the same effect is not observed in endotherms with internal temperature regulation [30]. In contrast, Liu et al. (2023) did not detect any correlation between growth temperature and mutation rate per site per year in 13 prokaryotes and 107 eukaryotes, suggesting that the effect of environmental temperature on mutation rate may not be universal [31].

Different populations can be exposed to different mutagens such as chemicals and pollutants that can interfere with replication and repair. Exposure to mutagens may increase the supply of new mutations and can consequently affect substitution rates, although the long-term outcome also depends on selection and genetic drift [32,33,34].

Exposure to radiation, including natural ultraviolet radiation, is a classic environmental mutagen that increases the occurrence of errors during repair and replication, increasing mutation rates. While there is no unequivocal study quantifying a linear relationship between global UV exposure and substitution rate across species, intense UV exposure is strongly linked to increased selective pressure and accelerated adaptation [35,36]. High natural background radiation in a coastal region of Kerala, India, was associated with higher mutation rates in human mtDNA than elsewhere [37]. Nevertheless, to our knowledge, there is no other study showing local alterations of mutation rates.

Purifying selection and positive selection can both substantially alter molecular-clock estimates by affecting the probability that mutations become fixed. Purifying selection removes deleterious mutations from populations, reducing the amount of genetic variation that contributes to long-term substitution rates. However, deleteriousness is not binary as many mutations are mildly deleterious or nearly neutral. As a result, there is a continuum between the instantaneous mutation rate, which includes newly arising variants that may still be segregating, and the long-term substitution rate, which is mostly composed of mutations that have survived the filtering effects of selection. This contributes to time-dependent molecular evolutionary rates, as discussed later on. In recombining systems, selection can act more directly on specific alleles, whereas in non-recombining systems selection affects the entire lineage carrying a deleterious mutation. These effects can make estimated molecular clocks appear accelerated or decelerated depending on the timescale considered, and may lead to over- or underestimates of divergence times if not properly accounted for, particularly when a rate is applied to a timescale different from that over which it was estimated.

This effect is particularly clear in human mtDNA. Younger human mtDNA clades contain a higher proportion of non-synonymous mutations [38,39]. Such mutations are more likely to be deleterious [40,41]. Young clades also contain a higher proportion of variants in structurally constrained tRNA and rRNA [42,43]. Beyond mtDNA, purifying selection is clear in autosomal SNPs altering amino acids [44]. This raises an important consideration for researchers. Researchers need to understand the functional aspects of the genetic system or variation under study, to determine whether the analysed variation may be under purifying selection. For example, Kivisild and collaborators [39], followed by Soares et al. [38], proposed that synonymous variation in human mtDNA provides a more nearly linear molecular clock because it is less affected by strong selection. In some cases, it is less clear; the fast-mutating control region of the human mtDNA was typically described as neutral in phylogeographic studies, but it contains several functional components for the replication and transcription of the mtDNA [45], including regions that may contribute to single-stranded secondary structures important for regulation [46].

Although the average effect of purifying selection across time can be modelled [38], variation in the strength of this effect can also influence molecular-clock estimates. Relaxation of selective constraints can make the clock faster as more slightly deleterious mutations persist in the population. Such relaxation has been suggested following human demographic expansions after the Last Glacial Maximum [47]. During rapid expansion, transient founder effects and increased drift may reduce the efficacy of purifying selection. By contrast, once populations attain a large effective size, purifying selection is generally expected to become more efficient because mildly deleterious mutations are less likely to drift to high frequency [48]. Conversely, small effective population size does not necessarily imply weaker selection in all cases, but increases the relative influence of genetic drift, making the fate of mildly deleterious mutations more stochastic and reducing the average efficacy of purifying selection. For example, Aizpurua-Iraola et al. (2025) reported that purifying selection was not relaxed in the human population of Eivissa, despite its reduced effective population size, when compared with other populations [49].

Positive selection can accelerate the evolution of a specific gene, making the molecular clock faster as more functionally relevant mutations are positively selected. Arbiza et al. (2006) found significant deviations from the strict molecular clock in hundreds of human and chimpanzee genes: The authors interpreted many of these genes as showing accelerated evolution [50]. However, one must consider that positive selection is rare when compared with purifying selection. For example, some earlier work on the distribution of non-synonymous mutations in human mtDNA was interpreted as evidence of regional effects of positive selection associated with different climates [51]. However, most amino acid changes failed to show geographic patterns and they were more likely to represent mildly deleterious mutations that were still undergoing removal by purifying selection [40].

A final demographic factor is population size. As noted above, effective population size influences the efficiency of both purifying and positive selection. By itself, population size should not directly determine the molecular clock under a strictly neutral model. However, small effective population sizes increase the stochastic role of genetic drift, potentially producing branches with fewer or more substitutions than expected under an average substitution rate. They can also reduce the efficiency of selection, allowing mildly deleterious mutations to persist or even become fixed. Population size can therefore influence molecular-clock estimates indirectly, by altering the balance between drift and selection and by increasing stochastic variation around the expected substitution rate.

### 2.4. Statistical and Methodological Factors

In addition to the biological factors that influence substitution rates, researchers must estimate these rates from molecular data. The same sequence dataset may yield different divergence-time estimates when analysed using different clock models or calibration priors. Unlike the factors discussed above, these statistical and methodological factors do not alter the underlying biological rate itself, but they can substantially affect the rate inferred from the data (the estimated molecular evolutionary rate). Rate estimates depend on the clock model; the analytical framework used to infer the phylogeny and branch lengths, such as maximum likelihood or Bayesian inference; the method used to count or model substitutions; the substitution model; data quality; and the calibration strategy. Substitution models are required because different nucleotide or amino acid changes do not occur with equal probability, and the same site may change more than once over evolutionary time. By accounting for these patterns, the models provide more accurate estimates of branch lengths and divergence times. Because these methodological choices determine how molecular variation is converted into evolutionary time, they form the central focus of the remainder of this review. The following sections therefore examine the main clock models, analytical frameworks, statistical tests and calibration strategies used to estimate evolutionary rates and divergence times and to assess clock-model adequacy.

## 3. Variations on Molecular Clocks

The concept of the molecular clock was originally formulated as a relatively simple relationship between divergence time and the accumulation of molecular differences. In this framework, genetic differences between lineages were interpreted as the result of substitutions accumulating through time at an approximately constant rate. However, the molecular-clock theory has evolved substantially, both conceptually and statistically, leading to several alternative models that differ in how they treat rate variation across lineages and timescales.

**Figure 2:**
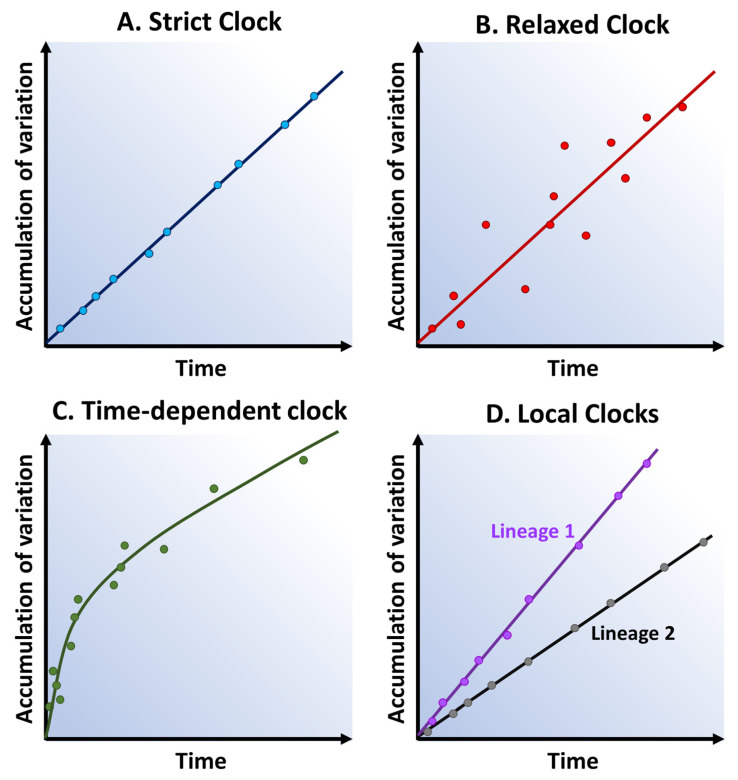
Schematic representation of four molecular clock models, illustrated by comparing the accumulation of molecular variation over time in hypothetical datasets. Points represent individual observations, while the lines indicate the expected relationship between accumulated variation and time. Panels correspond to (**A**) a strict clock, shown in blue; (**B**) a relaxed clock, shown in red; (**C**) a time-dependent clock, shown in green; and (**D**) local clocks, with grey and purple indicating two sections of the phylogenetic tree evolving at different rates.

**Figure 3:**
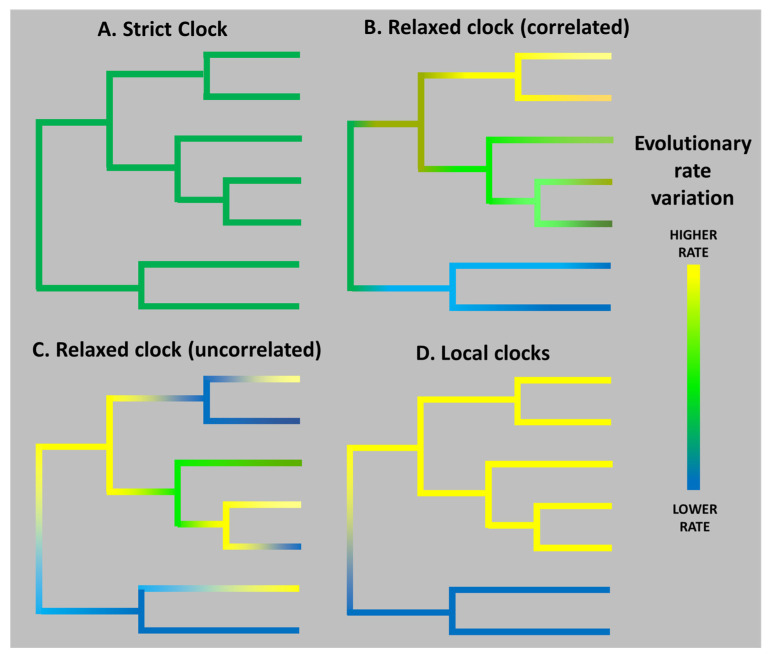
Molecular evolutionary rate variation across four molecular-clock models. Panels correspond to (**A**) a strict clock, (**B**) a correlated relaxed clock, (**C**) an uncorrelated relaxed clock and (**D**) local clocks.

The original molecular-clock model corresponds to what is now called a strict molecular clock (Figure 2A and Figure 3A). It assumes a constant rate of molecular evolution across all lineages and time. This model may be appropriate for a particular genomic region within a species or among closely related species, especially when rate variation among lineages is limited. However, the assumption becomes increasingly restrictive in deeper evolutionary comparisons, where lineages may differ substantially in life-history traits, demographic histories, selective pressures and environmental conditions. Even closely related lineages may exhibit evolutionary rate variation for the reasons discussed above. Despite these limitations, strict molecular clocks can be statistically adequate in many datasets [52,53], including widely studied mtDNA clocks in humans and other mammals [41,54,55] where likelihood-ratio tests have often failed to reject clock-like behaviour [56]. However, failure to reject a strict clock does not demonstrate that all lineages evolve at exactly the same rate; it only indicates that the observed branch-length variation is compatible with the model given the available data. In contrast to the strict clock, relaxed molecular-clock models allow molecular evolutionary rates to vary among branches of a phylogenetic tree (Figure 2B) [57,58,59]. This is considered more realistic as different lineages and species may evolve at different rates owing to the biological, demographic and environmental factors discussed above, as selective pressure, temperature and population size. Relaxed-clock models are commonly divided into two broad classes: correlated (Figure 3B) and uncorrelated relaxed clock (Figure 3C). In the latter, evolutionary rates are obtained from a statistical distribution independently of the position of the lineage in the phylogeny. In the case of correlated relaxed clocks, evolutionary rates on related lineages or clades are more similar than rates in phylogenetically distant lineages, assuming that evolutionary rates are autocorrelated between ancestral and descendant branches. Relaxed clocks are particularly useful in deep phylogenies, where the analysis includes species or lineages with different substitution rates, potentially reflecting differences in mutation processes, selection and life-history traits.

However, the use of relaxed clocks can be problematic. Because they estimate additional parameters [60], they can be sensitive to small datasets, limited temporal signal or poor-quality sequence data. Distinguishing among competing relaxed-clock models can also be difficult, for example using Bayes factors [61], particularly when datasets are small or weakly informative, increasing the risk that model choice becomes arbitrary. Strong bias may be introduced if the assumed rate distribution does not adequately represent the true pattern of the evolutionary rate variation. Because relaxed-clock models are highly flexible, sequencing errors, alignment problems or misplaced taxa may sometimes be absorbed as apparent branch-specific rate variation rather than identified as data-quality problems. This can create false confidence in divergence-time estimates if data quality and model adequacy are not carefully assessed [62].

Time-dependent molecular rates represent a different dimension of rate variation from the among-branch heterogeneity accommodated by relaxed-clock models (Figure 2C). Relaxed clocks allow substitution rates to vary among branches of a phylogenetic tree, whereas the time-dependent rate phenomenon refers to systematic differences among molecular evolutionary rates estimated over different temporal depths. Time-dependent clocks refer to the fact that estimated molecular evolutionary rates depend greatly on the timescale over which they are estimated and later applied. Evolutionary rates are often much faster when based on short timescales, such as pedigrees or recent events, than the substitution rates obtained over ancient evolutionary timescales, often from fossil calibrations. Accordingly, variation may accumulate rapidly over recent timescales and more slowly over deeper timescales, producing the nonlinear relationship shown in Figure 2C. When the estimated molecular evolutionary rate itself is plotted against temporal depth, this time-dependent pattern is often described as J-shaped. The main reason for this pattern is likely the previously explained effect of purifying selection. At short timescales, many deleterious mutations may still be segregating, whereas strongly deleterious mutations are rapidly eliminated and mildly deleterious mutations may take longer to be removed [38,40,41,43].

Evidence for time-dependent rate variation was reported in a study on Adélie penguins, where ancient DNA (aDNA) calibrations produced estimated molecular evolutionary rates six times faster than fossil calibrations [63], although previous analyses failed to detect such a trend [64]. Time-dependent clocks have been documented in a wide range of systems, including viruses [65], bacteria [66], Chloroplast DNA in plants [67] and different genes in humans [68]. There are two main approaches to developing a time-dependent molecular clock: one uses different calibrations at different timescales; the other uses a class of mutations that is neutral, or close to neutral, and not extremely fast-evolving, in order to avoid recurrences and hidden mutational events, and then models the contribution of slightly deleterious mutations at different timescales of the tree. This was our approach with human and other mammalian mtDNA, where we used synonymous variation for this purpose [38,41]. This makes it possible to estimate a time-dependent curve even when intermediate calibration points are difficult to obtain [68].

Finally, the local clock (Figure 2D and Figure 3D) is an extension of the molecular clock hypothesis that permits substitution rate constancy within specific phylogenetic clades, as in a strict clock, but allows different clades to have different substitution rates [69,70,71]. A local clock divides the tree into user-defined partitions, for example clades or branches, each with its own constant substitution rate. The local clock can theoretically approximate the use of correlated relaxed clocks (Figure 3B), in the sense that it allows specific clades to have more similar substitution rates. Correlated relaxed clocks do not require the user to specify in advance which lineages share a similar rate; however, local clocks offer results that are easier to interpret biologically when prior knowledge strongly suggests evolutionary rate shifts at certain phylogenetic splits. They also offer a simpler model, avoiding some drawbacks of relaxed clocks, as discussed above, when there is strong evidence for evolutionary rate homogeneity within partitions. Local clocks have been successfully applied within primates [71], flowering plants [72] and viruses [69]. In fact, this last example used an approach called flexible local clocks, which allowed local strict clocks in some clades and relaxed clocks in others [69].

## 4. Analytical Tools for Estimating a Molecular Clock and Statistical Evaluation

In this section, we provide a brief overview of tree-building methods and models of sequence evolution, focusing on how they can influence molecular-clock estimation and application.

### 4.1. Tree-Building Approaches and Branch Length Estimates

Different methods can be applied to phylogenetic reconstruction when the objective is to estimate or apply a molecular clock. Character-based phylogenetic methods allow the reconstruction of mutational events within a given sequence across species or individuals. These methods include ML, BI and maximum parsimony (MP). Each method has strengths and limitations in the context of molecular-clock analyses.

ML methods estimate the tree that maximizes the probability of observing the sequence data given a specific evolutionary model. Branch lengths are estimated in terms of expected substitutions per site, which is necessary for the estimation and application of a molecular clock. ML accommodates different substitution models and evolutionary rate heterogeneity across sites, and it can be extended to handle partitions when heterogeneous evolutionary processes apply to different sections of the dataset, namely different genes in a concatenated alignment or different codon positions in protein-coding DNA sequences. The substitution model employed influences the estimated rate of recurrent mutations in the data, which directly affects branch-length estimation. ML can be run under a strict clock or without a clock constraint, in which case branch lengths are estimated freely in substitutions per site. Without temporal calibration, however, branch-specific evolutionary rates cannot be separated from branch durations. This is an important feature for testing the applicability of a molecular clock. ML also generally allows the implementation of local clocks. It is computationally efficient and robust for large datasets, but it typically assumes a single final topology, failing to account fully for uncertainty in tree reconstruction [73]. Software tools, such as PAML [74], IQ-TREE [75], HyPhy [76] and PhyML [77], offer various possibilities for the estimation, application and statistical testing of molecular clocks using ML.

BI methods use Markov Chain Monte Carlo (MCMC) sampling to estimate the posterior distribution of trees, branch lengths and model parameters, including molecular-clock parameters, based on the sequence data. Uncertainty in topology and branch length is intrinsically incorporated in BI methods, making them a frequently preferred approach for molecular-clock dating. BI methods are highly flexible, allowing the implementation of strict and relaxed clock models, the simultaneous estimation of divergence times and phylogeny, and the application of multiple calibration points while accounting for their uncertainty. Partitions can also be applied in BI methods. BI inference is computationally intensive and can handle large datasets; however, the analyses are often difficult to evaluate. Because topology, evolutionary rate variation and model parameters are estimated simultaneously based on various priors, the model can fit the data in ways that do not necessarily reflect biological reality. In particular, BI estimates can be strongly influenced by prior assumptions on substitution rates, clock models, tree priors and fossil calibrations, especially when molecular data and calibrations are weakly informative. In such cases, posterior estimates may be driven more by the priors than by the sequence data [78,79], yielding precise but biased age and evolutionary rate estimates [80]. The fact that BI is often regarded as a gold standard in molecular-clock estimation may also lead to overconfidence by the researcher and discourage alternative analyses. BI molecular-clock estimates can be performed using tools such as MCMCTree [81], MrBayes [82] and BEAST [83], which offer multiple model and calibration options.

MP aims to reconstruct the tree that requires the minimum number of evolutionary changes. MP is highly intuitive and computationally less demanding. However, because it does not explicitly model sequence evolution or branch lengths, it is often regarded as less suitable for molecular-clock estimation. One important limitation is that MP does not fully account for recurrent and hidden mutations in the phylogeny, and consequently branch lengths can be underestimated. Another issue is related to the reconstruction itself, as long-branch attraction can cause unrelated lineages to be inferred as more closely related due to convergent substitutions. This phenomenon is more frequent in longer branches, which are often used for molecular-clock calibration, and can again lead to branch-length underestimation. However, MP has been a useful tool in phylogeography [84] and in intraspecific phylogenies where recurrent mutations and reversions have low probabilities. MP networks are particularly useful in phylogeography because they often deal with closely related haplotypes within species or populations, where recurrent mutations are relatively rare and ancestral or intermediate haplotypes may still be sampled. In these cases, networks provide an intuitive representation of mutational steps, unsampled intermediates and geographic structure without forcing all relationships into a strictly bifurcating tree. Haplotype networks can also be intuitively employed in scenarios where ancestral sequence types are common.

In MP, branch length can be intuitively estimated as the number of mutations in each branch. This leads to measures such as ρ (rho) [85]. The ρ statistic is a simple molecular-dating estimator that measures the mean number of mutations separating sampled sequences within a clade from their inferred ancestral root. When scaled by an appropriate evolutionary rate, it provides an estimate of the clade’s age. A key advantage of ρ is that it is mathematically unbiased under standard mutation models and does not require a fully parametric demographic model or tree prior, making it a robust baseline estimator against which more model-dependent approaches can be compared, despite its comparatively high variance. As demonstrated analytically and empirically, ρ yields age estimates that are broadly consistent with ML and BI methods, while its transparency and limited parameterization reduce sensitivity to overfitting and prior misspecification, particularly in well-resolved phylogenies based on high-quality sequence data [86].

### 4.2. Statistical Methods to Evaluate the Reliability of a Molecular Clock

It is possible that some molecular datasets may be incompatible with a strict molecular-clock model. Substitutions may accumulate at different evolutionary rates across lineages owing to differences in life-history traits, selective pressures, or episodes of lineage-specific rate alteration, among others. Before applying a molecular clock, it is therefore necessary to evaluate whether the data and calibrations contain sufficient information to estimate evolutionary rates and times; for heterochronous datasets, this includes testing for temporal signal. The molecular-clock assumption should not be adopted automatically, but treated as a hypothesis to be tested before being used for evolutionary interpretation. A strict-clock model may be rejected for a dataset, while a relaxed or local clock may still provide an adequate description.

Before evaluating a molecular-clock model, the underlying sequence data should be carefully screened. Alignment quality should be assessed, poorly aligned regions removed, substitution saturation evaluated, and recombination excluded or minimised where possible. Sequence quality, taxon sampling and model specification should also be checked. Assuming that these preliminary steps have been completed, the next stage is to assess temporal signal and clock-model adequacy. An initial step is to conduct exploratory analyses. One can run an MP tree or network, or an ML tree with no clock imposed. Branch lengths may naturally vary substantially among clades or samples. For example, in the human mtDNA phylogeny where a strict clock is often accepted [38], the African haplogroup L2 has shown a very divergent branch length in one of its branches [87,88]. Such an approach is also useful to detect possible outliers, namely low-quality sequences whose removal would normalize the use of a molecular clock.

Particularly in the case of heterochronous datasets such as viral and bacterial sequences, root-to-tip regression can be applied to test whether genetic divergence increases with sampling time, using software tools like TempEst [89]. It is important to define that this does not provide a formal statistical validation or rejection of the molecular clock, but allows a first diagnostic test.

Other diagnostic tests are relative rate tests, such as Tajima’s test [90]. This is not a formal evaluation for a full dataset but allows testing wether two sequences show the same evolutionary rate in relation to an outgroup. The analysis can be used to develop a quick screening [91] and even detect outliers in the two-by-two comparisons. Simple chi-square clock tests can be used as relative-rate tests to check whether two lineages have accumulated the same number of substitutions since their divergence from a common ancestor.

One of the most common and statistically robust approaches to test a strict molecular clock is the likelihood-ratio test (LRT) [92], which is implemented in PAML [74] and HyPhy [93]. Basically, LRT compares the maximized log-likelihood of a clock-constrained model with that of an unconstrained model in which branch lengths vary freely (no-clock). This likelihood-ratio statistic is compared against a chi-square distribution with degrees of freedom approximately equal to the difference in number of parameters between the two models. This allows the rejection or non-rejection of a global molecular-clock hypothesis. The LRT has been applied to the widely used human mtDNA molecular clocks either aimed at the complete genome or coding region [38,41,54,94,95,96] consistently failing to reject the clock hypothesis. However, this is not an absolute rule, as observed for the African haplogroup L2 where LRT rejects the clock model [87,88,97]. Additionally, the LRT may detect very small deviations from a strict molecular clock in large genomic datasets, so statistical significance should be interpreted alongside effect size and biological context [52].

Likelihood-ratio tests have also been used to compare global-clock and local-clock models [98], although their validity for local-clock comparisons can be limited depending on model specification [99]. For local-clock comparisons, a permutation-based approach was proposed that avoids these statistical caveats [70].

However, as mentioned before, the rejection of the strict molecular clock does not necessarily imply that a clock is inappropriate; it can mean that a relaxed or a local clock is necessary. As defined before, relaxed-clock models allow evolutionary rates to vary among branches, whereas local-clock models assign different rates to predefined clades or lineages.

Strict vs. relaxed clock comparison is usually performed in a Bayesian setting using BEAST [83] or BEAST2 [100], where marginal likelihoods or posterior support for different clock models are analysed. Bayes factors are commonly used in BEAST to compare clock models [101], and model averaging is sometimes recommended when no single relaxed-clock model is clearly preferred. In a model-averaging approach, information across a set of plausible models is combined, weighting each one by its support, so uncertainty about model choice is carried into the final inference [102,103]. For Bayes factors, path sampling and stepping-stone sampling are recommended over the harmonic mean estimator, which is known to be unstable for marginal-likelihood estimation. Beyond model selection, posterior predictive simulation can assess whether a clock model is adequate, meaning whether it can generate data similar to the observed data, not just whether it fits better than alternatives [61].

A contrasting view is that Bayesian model comparison relies on marginal-likelihood estimation and Bayes factors, which are sensitive to prior specification and are less naturally framed as hypothesis-falsification tests than likelihood-based clock tests [56,104].

For heterochronous sequence data, date-randomization tests (DRT), or tip-date permutation tests, can be used. The basic idea is to compare the clock-rate estimate from the real sampling dates with estimates obtained after randomly permuting dates among sequences; if the real-data estimate is clearly separated from the randomized distribution, the dataset is considered to contain sufficient temporal signal to support molecular-clock inference [105]. This approach is widely used in viral evolution [105], bacterial genomic epidemiology [106] and aDNA. Methods include TreeTime for ML-based assessment [107], LSD2 for least-squares dating [108] integrated in IQ-TREE [109], and BactDating for bacterial datasets [106]. A more recent Bayesian approach is the Bayesian Evaluation of Temporal Signal (BETS), which compares models with real dates, randomized dates, and no dates to formally assess temporal signal. BETS is particularly useful when DRTs give ambiguous results or when sample sizes are small [110]. When no temporal signal is detected, molecular dating may not be feasible with the current data.

## 5. Calibration of the Molecular Clock

Irrespective of the model or methodological approach used to estimate branch lengths, uncalibrated phylogenetic trees provide, at most, relative measures of evolutionary divergence. A molecular clock becomes informative for absolute dating only when these relative distances are anchored to an external temporal framework through calibration.

**Figure 4:**
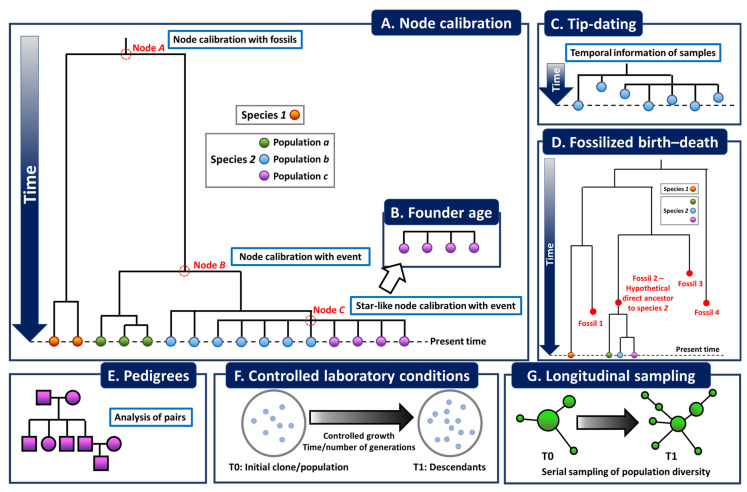
Various approaches to calibration including node calibration (**A**), founder ages (**B**), tip dating (**C**), fossilized birth-death process (**D**), pedigrees (**E**), controlled laboratory conditions (**F**) and longitudinal sampling (**G**).

In practical terms, calibration is the procedure that allows branch lengths, which are initially estimated as genetic distances or expected numbers of substitutions, to be translated into absolute time depths. This is achieved by anchoring the tree to an external temporal reference, such as a fossil age, dated sample, biogeographic event, archaeological date, or another external temporal reference (Figure 4). Once such a temporal anchor is introduced, the molecular clock can convert relative genetic divergence into estimates of evolutionary time. In this section we will review the basic approaches to calibration.

### 5.1. Node Calibration

Node calibration is one of the earliest and most widely used approaches, particularly when fossil constraints are available. This is the classical approach for dating deep evolutionary divergences. Basically, a fossil is assigned to a specific lineage or clade and its geological age is used to constrain the age of a node, assuming a split time between those lineages, usually species. Such is the case for Node A in Figure 4A. Assuming an age for the specific split allows a time-frame to be applied to the whole phylogeny. The literature is rich in this type of calibration, namely using the Human–chimpanzee split [38,111] or the deeper Old World monkeys vs. hominoids [112], the Mammal–bird divergence [113,114], Mouse–rat split [113], Eutheria–Metatheria [114], and the angiosperm crown-group appearance in the fossil record for dating plants [115,116,117]. Traditional fossil-node calibrations are somewhat abundant for animals and plants, but comparatively scarce for microorganisms because the microbial fossil record is limited and often difficult to assign confidently to specific groups.

Node calibration with the paleontological record has some drawbacks, namely the fact that fossils provide minimum ages rather than exact divergence times, because the oldest known fossil of a clade represents only a proximal estimate and is likely an underestimate of the true node age. Nevertheless, this is less problematic if we consider the time scale. For example, if a clade is assumed to be 50 million years old (Myr) based on the oldest fossil but is actually 55 Myr according to unknown evidence, the error is about 10%, which might not be drastic given the broad confidence intervals typically associated with node estimates. Additionally, fossil calibrations are commonly applied as soft bounds that permit a small probability of violation, rather than hard bounds that strictly enforce minimum and maximum ages, to accommodate uncertainty in the fossil record.

Other problems associated with node dating include the presence of ancestral polymorphisms. Before the split of a species into two diverging lineages, ancestral individuals or populations may already carry genetic diversity, causing gene coalescence times to predate the speciation event. In Bayesian analyses, fossil calibrations are most often implemented as node-age priors; given the possibility of applying multiple calibration points under relaxed-clock models, the posterior distribution of node ages can then recalibrate these priors, effectively allowing the data to update the fossil-based constraints [41]. Another issue associated with deep evolution is the time-dependent nature of evolutionary rates, whereby long-term substitution rates are typically slower than short-term molecular evolutionary rates estimated within recent clades, likely because purifying selection gradually removes deleterious mutations over time.

Another mode of node dating in calibration is to associate the age of a clade with a specific geological, climatological or biogeographic event. This calibration can correspond to the split time of species or just the separation between groups or populations within the same species (as Node B in Figure 4A). In the absence of fossil records, these events can be assumed and defended for a given split.

The emergence of islands provides one such calibration. Examples include the sequential formation age of the Hawaiian Islands, which has been used to calibrate divergences in endemic plants and arthropods, under the assumption that colonisation occurred soon after island formation [118], and the emergence of the Macaronesian islands, which has been applied to plant groups, marine invertebrates and lizards [119,120,121]. Changes in terrestrial connectivity and marine barriers provide another class of calibration. The formation of the Isthmus of Panama connected North and South America, enabling the large-scale fauna exchange [118] while simultaneously separating Caribbean and Pacific marine species populations [122]. Postglacial land bridges to Scandinavia have similarly been used in phylogeographic calibration [123]. Seaway dynamics include the closure of the Central American Seaway [118,124] and the opening and closure of the Strait of Gibraltar associated with the Messinian Salinity Crisis [125].

Larger-scale tectonic processes, including the breakup of Gondwana and other plate-tectonic events, have been used to calibrate deep divergences [126]. Mountain uplift, such as the rise of the Andes and the Himalaya–Hengduan Mountains, can create both dispersal barriers for biota and new high-elevation habitats [127,128,129,130]. The formation of ancient lakes, including Lakes Tanganyika and Baikal, has been used to calibrate endemic radiations within those lakes [131]. Finally, past climate events, including postglacial sea-level changes and glacial–interglacial cycles, have frequently been used in phylogeographic studies of species whose distributions expanded or contracted with ice sheets and sea-level fluctuations [118,123,132,133,134,135], including studies of human migrations [136,137]. As with fossil calibrations, node calibration using geological, climatological, or biogeographic events is constrained by minimum-age uncertainty and the potential effects of ancestral polymorphism that could be even more drastic for more recent calibration points as often used in this type of calibration. However, it also depends critically on a robust and defensible match between the biological event and the external historical assumption used for calibration points.

A related form of node calibration uses star-like phylogenetic patterns that may reflect rapid population expansion, as illustrated by node C in Figure 4A. The logic is that a demographic or environmental event may have triggered a major expansion, leaving a shallow, poorly structured clade with many singleton mutations rather than deep internal branching [138]. This differs from calibrating a split between two lineages: here, the focus is on estimating the timing of the expansion itself, often by placing the inferred date just before or around the onset of rapid growth. Such correlations have been made most often with past climatic changes, especially postglacial expansions in Europe [139]. A more statistical analogue of this approach is the identification of expansion times from Bayesian skyline plots. One example of this approach concerns the human mtDNA haplogroups H1 and H3 [139,140], which show highly star-like phylogenies in Europe and were traditionally interpreted as signatures of postglacial expansions. This interpretation has also been used as a prior in Bayesian calibration [141]. However, aDNA evidence and more recent founder-age estimates suggest that the main expansion most likely occurred during the European Neolithic rather than immediately after the Last Glacial Maximum [142,143].

### 5.2. Founder Calibration

A related but less commonly applied approach to calibration is the estimation of founder ages. Unlike traditional node dating, this method focuses on the timing of a colonisation event by comparing the diversity found in a sink population with that in a source population for a specific clade (Figure 4B). The underlying principle is that private diversity in the sink arose after migration, which effectively resets the molecular clock to zero once the founding lineage is identified. While this approach is typically used to date migration events and the impact of a given migration based on an existing molecular clock [144,145], it can also be employed as a calibration point. Island colonisation studies in birds and insects have often inferred colonisation timing from the number of private mutations in island populations relative to the mainland, under the principle that the molecular clock effectively resets at colonisation [146,147]. For a direct estimation of a clock based on private diversity, we calibrated a Y-STR haplotype network of haplogroup C-M208 by assuming that the private diversity present in Remote Oceania, a well-established migration context, accumulated after the colonisation events that began roughly 3000 years ago [148]. In other contexts, such founder age estimates have been used primarily as hypothetical checkpoints for calibration, as we will discuss below, although these could have also been used for calibration [149,150].

### 5.3. Tip Dating

Tip-dating (Figure 4C) is a molecular dating and calibration approach that treats the ages of sampled sequences (or fossils) as explicit data points, placing them directly on the tips of the phylogenetic tree rather than using them only to constrain internal nodes [151,152]. In this framework, the known or estimated ages of the sampled individuals serve as calibration information that is integrated into the tree inference itself, allowing the model to simultaneously estimate phylogeny, divergence times, and evolutionary rates. This approach is particularly powerful for time-sampled datasets such as rapidly evolving viruses, where sequences are collected over a few years or decades, and for palaeogenomic studies that combine aDNA with modern genomes.

Tip-dating has become an important approach in bacterial and viral phylogenetics because many viral and bacterial datasets are sampled over short enough time spans that collection dates contain genuine temporal information [153]. In HIV-1, tip-dating has been used to infer the timing of viral lineage evolution and proviral integration events, with sampling dates serving as explicit tip calibrations [154,155]. During the COVID-19 pandemic, tip dating was widely used to date the emergence of SARS-CoV-2 and its subclades [156,157,158]. For dating bacterial genomic phylogenies, BactDating has become a standard approach and has been applied mainly to pathogenic bacteria [106,159,160,161].

Tip-dating has become a central approach in palaeogenomics, where the known ages of aDNA samples are used as explicit tip calibrations to infer time-scaled phylogenies alongside modern sequences. In this framework, the sampling dates of ancient genomes are treated as data, allowing the model to simultaneously estimate phylogeny, divergence times, and evolutionary rates without relying solely on node constraints from fossils or biogeographic events [162,163,164,165]. This approach is particularly powerful for taxa with scarce fossil records, such as humans and extinct megafauna. For example, tip-dating has been applied to large datasets of ancient and modern human genomes to estimate evolutionary rates and past demography mostly using human mtDNA [148,166,167,168,169].

Tip-dating is powerful, but it has several important drawbacks. First, it requires that the dataset contains a clear temporal signal: the relationship between root-to-tip distance and sampling time must be strong enough to justify using sampling dates as tip calibrations. If temporal signal is weak or absent, the dates associated with sampling times can still bias the inferred tree and distort evolutionary rate estimates. Several methods exist to assess temporal signal before tip-dating, including root-to-tip regression (e.g., TempEst [89]), DRTs [105], and BETS [110], which compare the fit of models with actual versus randomized sampling dates to determine whether the data contain sufficient temporal signal as discussed above. Second, tip-dating is sensitive to date-randomization problems: if the sampling dates are not informative (as when sequences are all collected over a very short interval or when the time span is too small relative to the overall depth of the tree), the model may produce spurious estimates that depend more on the model assumptions than on the data [105]. Third, standard tip-dating models often assume that evolutionary rates are relatively constant or follow a well-specified relaxed-clock model, which can be problematic in fast-evolving pathogens. Also, time-dependent molecular evolutionary rates can distort tip-dating estimates [65], particularly when the sampling interval is short relative to the tree depth. This time-dependence is strongly driven by purifying selection, which removes deleterious mutations over time, leading to higher short-term rates that reflect recent mutations with little selection effect (typically associated with clades containing dated tips), while deeper time frames show lower rates due to the larger effect of purifying selection [166]. This typically leads to overestimates of the evolutionary rate. Fourth, tip-dating with aDNA introduces additional challenges because aDNA is often fragmented, contaminated, and has uncertain calibration dates, which can lead to misplacement of ancient samples and bias age estimates if their ages are inaccurate. Additionally, aDNA likely contains artefacts that will increase the branch length of the dated tips, which could again overestimate the molecular clock. Considering that branch lengths are small in many cases where tip-dating has been used (as in the human mtDNA molecular clock), a small number of artefacts can distort the clock into providing faster evolutionary rates. Recently, Fernandes et al. (2025) [170] showed that the Allen Ancient DNA Resource (AADR), one of the most comprehensive resources for ancient human palaeogenomics, contains several nonsense and missense mutations that are likely to be highly pathogenic, with nonsense mutations over 70 times more frequent in aDNA than in modern samples. Fifth, tip-dating can be computationally expensive and convergence can be slow, especially in large datasets with many time-stamped sequences, making it difficult to obtain robust Bayesian posterior estimates without extensive runtime.

Interpreting tip-dated trees can be challenging because the model simultaneously estimates phylogeny, node ages, and evolutionary rates, and disentangling the relative influence of data, model specification, data quality and priors often requires careful diagnostics and multiple sensitivity analyses.

### 5.4. Fossilized Birth–Death and Total-Evidence Dating

The fossilized birth–death (FBD) process provides a model-based framework for incorporating fossil evidence into divergence-time estimation in a different way from node dating. It models speciation and extinction processes but also the preservation and sampling of lineages through time (Figure 4D). In a implified formulation, estimated parameters include speciation rate, extinction rate, fossil-sampling rate and the probability of sampling extant taxa. Fossils and extant specimens are treated as samples generated by the same underlying diversification and sampling process. Fossils can be considered extinct terminal lineages but may also represent sampled ancestors, either of an extant lineage or of a later fossil [171,172,173]. This differs from node calibration, where fossils are translated into minimum ages or probability distributions placed on predefined internal nodes. Rather than using fossils only to constrain a small number of nodes, the FBD framework can incorporate a larger proportion of the fossil record and explicitly model the process through which fossil observations were generated.

The FBD analysis can be applied in different ways depending on the available evidence. In one approach, fossils are assigned a priori to particular clades on the basis of previous taxonomic or morphological evidence, and their ages contribute to divergence-time estimation even when morphological character matrices are unavailable [172]. A more integrative application is total-evidence dating, in which molecular sequences from extant taxa are analysed together with morphological characters scored for both extant lineages and fossil taxa, and with the stratigraphic ages of the fossils [174,175,176]. In this framework, fossil placement is also not necessarily fixed in advance but is estimated from the morphological evidence, meaning that phylogenetic relationships, divergence times, evolutionary rates and aspects of the diversification process of the lineages are inferred simultaneously with separate evolutionary models typically applied to molecular and morphological components. Total-evidence dating under the FBD process has several potential advantages. It can use multiple fossils rather than only the oldest fossil assigned to each calibrated clade and can permit fossils to occupy stem or crown positions according to the character evidence. Applications to groups such as Hymenoptera and penguins have shown that including fossil morphology can substantially affect both fossil placement and estimated divergence times [174,175,176].

These approaches also introduce important assumptions leading to potential sources of error. Fossil placement depends on the availability and quality of morphological characters and on the adequacy of the model used to describe morphological evolution. The construction and scoring of morphological matrices require considerable taxonomic and anatomical expertise and may involve subjective decisions concerning character homology, delimitation of character states, ordering, dependency, and the treatment of ambiguous or inapplicable observations. These difficulties are amplified by the fragmentary and uneven preservation of fossils, which often results in extensive missing data and restricts scoring to a subset of anatomical structures. Even outside total-evidence dating, misclassification of fossil or extant specimens can bias FBD-based estimates. Constant speciation, extinction and fossil-recovery rates may also be unrealistic for groups with long and complex evolutionary histories, although skyline FBD models allow these parameters to vary across temporal intervals [177]. FBD and total-evidence approaches should therefore not be regarded as automatically superior to node dating; their reliability depends on the quality of the fossil and morphological data, realistic sampling assumptions, appropriate prior specification, and sensitivity analyses under alternative models [175,177].

The FBD process and total-evidence dating are implemented in several Bayesian phylogenetic platforms. In BEAST2, sampled-ancestor and FBD analyses are available through associated packages, while morphological and molecular partitions can be incorporated into joint Bayesian analyses [100,173,176]. RevBayes provides a flexible graphical-model framework for specifying FBD and combined-evidence analyses, including models based on individual fossil occurrences or stratigraphic ranges [178].

### 5.5. Direct Measurement of Mutation Rate

Direct measurement of mutation rates involves observing mutations over known, very short time intervals, typically through pedigree studies (Figure 4E) and laboratory experiments (Figure 4F). These methods mainly provide germline mutation rates (the rate at which new mutations arise in a single generation) rather than evolutionary/substitution rates (the rate at which mutations accumulate over long evolutionary timescales). As discussed above, these two rates are related because variants emerge through germline mutations. However, comparing these rates is challenging because pedigree-based mutation rates may be higher than long-term evolutionary rates, sometimes substantially so, although the magnitude and even the presence of this discrepancy depend on the molecular marker and mutational process considered. Moreover, pedigree-based mutation rates are generally expressed per generation, whereas long-term substitution rates are often expressed per year; numerical comparisons therefore require conversion using an assumed generation time.

One such example is parent-offspring (pedigree) studies for Y-chromosome mutation rates. Various studies analyse father-son pairs to count Y-chromosomal short tandem repeat (Y-STR) mutations transmitted across a single meiosis. While these studies report mutation rates averaging 2–3 × 10^−3^ mutations per locus per generation [179,180,181], evolutionary rates inferred from population divergence are substantially lower at 6.9 × 10^−4^ mutations per locus per generation (assuming a generation time of 25 years in the publication) [182]. This discrepancy possibly arises because STRs mutate rapidly via slippage, but many mutations are lost over time due to selection or drift, creating a difference between observed germline rates and long-term substitution rates.

Mother–offspring pairs have also been analysed to count mtDNA mutations transmitted across a single generation. Pedigree-based estimates of the mtDNA mutation rate, obtained from mother–offspring transmission or deep maternal genealogies, are often substantially higher than long-term phylogenetic substitution rates [183]. In mtDNA, heteroplasmy, the germline bottleneck, drift, saturation and purifying selection can contribute to this difference, making pedigree rates inappropriate as direct calibrations for deep evolutionary timescales without careful modelling.

Studies based on whole-genome sequencing of parent–offspring trios or quartets have estimated de novo mutation rates of approximately 1–2 × 10^−8^ mutations per nucleotide per generation [184,185]. Assuming a generation time of 25 years, this corresponds to approximately 4–8 × 10^−10^ mutations per nucleotide per year, which is broadly comparable to long-term phylogenetic estimates of approximately 1 × 10^−9^ substitutions per nucleotide per year obtained from large-scale primate genomic comparisons [186,187], although rates vary among genomic regions and primate lineages. Nevertheless, a study of a large non-recombining region of the Y chromosome likewise found overlapping pedigree-based germline mutation-rate and phylogenetic substitution-rate estimates [188]. This contrasts with the substantially higher pedigree-based rates commonly observed for rapidly evolving markers such as mtDNA and Y-STRs. Nuclear whole-genome estimates therefore do not show the same marked discrepancy between pedigree-based and phylogenetic rates and may represent a case in which the germline mutation rate provides a useful approximation to the long-term evolutionary rate. However, given the complexity of whole-genome analyses and differences among studies in genome annotation, variant filtering, and the genomic regions analysed, these estimates may not be directly comparable, and their relationship remains an area of ongoing investigation.

These analyses can also be performed on deep-rooted pedigrees that include many more meiotic events and provide greater temporal depth [189]. Nevertheless, deep-pedigree rates still reflect germline mutations, not long-term substitution rates. Pedigree-based mutation-rate estimates are not restricted to humans; they have also been obtained in domesticated and model organisms such as dogs, wolves, rhesus macaques, and chickens, often using extended pedigrees or parent-offspring trios [190,191,192,193].

Pedigree-based mutation rates introduce an additional difficulty when used for molecular dating: they are usually estimated per generation, whereas evolutionary timescales are expressed in years. Conversion therefore requires an assumed generation time. In humans, this value is not fixed and has been widely debated, with estimates depending on whether one uses ethnographic, demographic, genealogical, or genomic information. Recent genome-based work has estimated an average human generation time of around 26.9 years across the last 250,000 years [194], but also shows sex-specific differences, with fathers generally older than mothers at reproduction. This means that even before considering selection, saturation or time dependency, the simple conversion of a per-generation mutation rate into a yearly rate can introduce significant uncertainty.

Another direct method for measuring mutation rates uses controlled laboratory experiments in organisms ranging from animals to microorganisms (Figure 4F). For example, studies in laboratory mice reported a germline base-substitution mutation rate of 5.4 × 10^−9^ per nucleotide per generation, about half the rate reported in humans [195]. Direct measurements have also been made for bacteria [196,197] and even viruses [198]. However, laboratory measurements of mutation rates may not translate directly to natural conditions because they are obtained under highly controlled, optimised environments that differ substantially from natural settings. Fitness, generation times and mutation rates may differ substantially between laboratory and natural environments. The mutation rate itself evolves rapidly in response to demographic and environmental changes. Thus, laboratory-derived mutation rates, while useful for controlled comparisons, may not reflect the true mutagenic processes operating in natural environments.

In fast-evolving systems, evolutionary rates can also be inferred from longitudinal sampling of the same population at multiple time points (Figure 4G), by tracking the accumulation of genetic diversity over time. This approach has been used, for example, in HIV-1, where serial samples from a single patient yielded a per-site per-year rate estimate based on the increase in polymorphism through time [199]. The very large number of SARS-CoV-2 genomes generated during the pandemic similarly enabled such approaches to be applied to the dating of population-level events, as the emergence and spread of genetic diversity could be tracked using phylogenetic methods in real time [200,201]. However, because these estimates depend on the fate of segregating variants, they are influenced by selection, drift and changes in population size. They therefore do not necessarily correspond directly to pedigree-based germline mutation-rate estimates. Their relationship with long-term substitution rates is also not straightforward, since short-term estimates may include transient variants that will not persist over longer evolutionary timescales.

### 5.6. Indirect Dating

When no direct external calibration is available, molecular dating can still be attempted by importing temporal information from outside the focal dataset, either through secondary node-age calibrations or through externally estimated substitution rates. These approaches can provide a temporal scale, particularly in BI frameworks where they may be implemented as priors, but they are intrinsically dependent on assumptions made elsewhere and should therefore be treated as sources of uncertainty rather than as fixed empirical anchors.

When direct external calibrations are unavailable, researchers sometimes rely on secondary calibrations: node ages taken from previous molecular dating studies and applied as temporal constraints in a new analysis. This approach can be useful when no fossils, dated tips, archaeological dates, or well-justified biogeographic events are available for the focal dataset. However, secondary calibrations are generally among the least robust forms of calibration, because they do not constitute independent evidence [202]. Any uncertainty, bias, model misspecification, taxonomic error, or inappropriate prior used in the original study can be carried forward into the new analysis, sometimes without being fully represented in the secondary constraint. This can create a chain of dependence among studies, where apparently independent molecular dates ultimately derive from the same original assumptions. For this reason, secondary calibrations should be used cautiously, ideally as broad priors rather than fixed ages, and their influence should be assessed through sensitivity analyses comparing alternative calibration schemes [203]. Nevertheless, in some cases they may be the only practical option for introducing a temporal scale into the analysis.

Another possibility, especially when no external temporal information is available for the tree itself, is to calibrate the molecular clock using an externally estimated substitution rate, often from an evolutionarily close species or from a comparable genomic region. In this case, the rate is not inferred from fossils, dated samples, or node-age constraints within the analysis, but is imposed from previous empirical work on the same gene, genome region, taxonomic group, or comparable evolutionary system. This strategy can be useful when independent evidence suggests that the rate is relatively stable and transferable. However, a rate estimated in one context may not apply to another, because evolutionary rates can vary among lineages, genomic regions, populations, demographic regimes, selective environments, and timescales. In Bayesian analyses, externally estimated evolutionary rates can be incorporated as priors on the clock rate, but their uncertainty should be explicitly represented and their effect on posterior date estimates should be evaluated through sensitivity analyses [79].

## 6. Historical Evaluation of Molecular Clock Results

It is possible that, even when a researcher approaches the estimation of a molecular clock properly (with appropriate models, well-inferred phylogenies, and reliable calibrations), the resulting clock estimates do not yield an easily interpretable picture for the data. This could reflect the fact that speciation and demographic events are not closely linked to known geological or palaeontological events, or it could indicate that the clock model itself is misspecified. Many factors can contribute to such discrepancies, including poorly dated fossils, high levels of ancestral polymorphism, evolutionary rate heterogeneity, or other sources of bias. Nevertheless, many molecular-clock estimates show good agreement with expectations from speciation patterns and demographic models. In the following sections, we first consider comparative phylogeographic examples involving diverse taxa. We then use human phylogeography as an extended case study, focusing principally on mtDNA because it provides a particularly well-characterised framework for evaluating time-dependent evolutionary rates, founder-age estimation and concordance with independent archaeological and ancient-DNA evidence. A complementary Y-chromosome example illustrates how molecular-clock interpretations can gain support from evidence generated after the original estimates. Finally, mycobacterial molecular clocks provide a contrasting microbial example and illustrate the risk of circular reasoning.

### 6.1. Phylogeographic Concordance

One way in which molecular-clock estimates can gain credibility is through concordance, a concept widely used in ecology and comparative phylogeography [204,205]. In this context, concordance refers to the observation that independently analysed taxa, genes, populations, or species assemblages produce temporally and geographically compatible histories. The logic is not that any single molecular date validates the clock, but that repeated agreement across independent lineages can support the inference that the clock estimates are capturing a real historical process. If several taxa distributed in the same region show demographic expansions, range shifts, or divergence events that fall within the same palaeoenvironmental window, and if these estimates were obtained using independent calibrations or plausible evolutionary rate models, then the convergence of evidence becomes informative. This form of evidence is especially useful when the events being inferred are not used as calibrations, because it avoids the most obvious form of circular reasoning.

A classic example comes from comparative phylogeography of Europe, where many temperate species show genetic signatures consistent with restriction to southern refugia during the Last Glacial Maximum followed by postglacial recolonisation of central and northern Europe. The phylogeographic patterns are not identical across taxa, because species differed in ecology, dispersal capacity, population size and refugial distribution. Nevertheless, studies comparing mammals, amphibians, insects and trees have found recurrent broad-scale patterns, including Iberian, Italian, Balkan and other southern refugial sources, followed by northward expansion after climatic amelioration [135,206]. However, concordance can easily lead to circular argumentation. In some cases, an independently calibrated molecular clock can be used to test whether a lineage expanded during the postglacial period, as in the brown bear example [207]. In other studies, however, the presumed timing of postglacial expansion is itself used to calibrate the clock [123]. In such cases, the resulting molecular date cannot provide independent support for the same historical event. As discussed above, some mtDNA haplogroups that were originally interpreted as having expanded during the postglacial period [137,139] and were later used as priors in calibration analyses [141] are now more likely to reflect later Neolithic expansions [142]. This illustrates the risk of assuming concordance between molecular dates and palaeoenvironmental events without independent validation.

The rise of the Isthmus of Panama is a particularly influential example, because it separated marine populations between the tropical eastern Pacific and western Atlantic, producing many transisthmian sister lineages. In principle, if divergences dated using independent fossil or phylogenetic calibrations cluster around phases of seaway restriction or final closure, this provides evidence that molecular clocks are recovering a shared geological signal. However, the Panamanian case also illustrates why concordance must be interpreted critically. Several studies have shown that transisthmian divergences are heterogeneous and that some estimates predate the canonical final closure of the Isthmus, suggesting earlier ecological or geological barriers, taxon-specific histories, continued gene flow, or problems with assuming a single vicariant event for all species pairs [208]. Fossil-calibrated analyses of marine invertebrates, including ark clams and earlier gastropod studies [209,210], recovered divergence times much older than final closure, while multilocus and comparative analyses of snapping shrimps, fishes and broader marine datasets have emphasised that different taxa do not necessarily record the same temporal history [211,212,213]. Thus, Panama provides a valuable example of partial concordance between molecular clocks and geological history, but also a warning that apparent agreement may depend on the calibration model and on whether the Isthmus itself is being used as the temporal anchor.

### 6.2. Human Phylogeography

Human demographic history provides one of the most intensively studied applications of molecular-clock inference. This is particularly true for mtDNA, which has been used for several decades to investigate the timing of major events such as the origin of the deepest maternal lineages, expansions within Africa, the dispersal of modern humans out of Africa, the settlement of Eurasia and Australasia, and later regional processes such as the recolonisation of Europe after the Last Glacial Maximum or the spread of farming. The appeal of mtDNA for this purpose is clear: it is non-recombining, effectively haploid, maternally inherited, present at high copy number, and structured into a well-resolved global phylogeny. These features make it possible to estimate the ages of haplogroups and founder lineages and to compare those estimates with archaeological, palaeoenvironmental and, more recently, aDNA evidence.

The time-dependent mitochondrial clock proposed by us in 2009 [38] was developed to address the problem of assuming a constant substitution rate, using a palaeontological calibration and a mathematical correction for time-dependency. The human mtDNA phylogeography framework therefore offers a particularly useful case study for asking whether a molecular clock can recover demographic events that are independently plausible from archaeology, palaeoenvironmental evidence and population history, while also avoiding the direct use of those events as calibration points. In the following examples, we therefore focus on cases where the human mtDNA clock has been used to infer demographic processes and where the resulting dates can be compared with independent lines of evidence. The purpose is not to argue that mtDNA alone can resolve human population history, especially in light of the resolution now provided by genome-wide aDNA, but to assess whether a well-characterised and explicitly time-dependent molecular clock can generate historically coherent demographic estimates.

The human mtDNA phylogeny coalesces at around 200 thousand years [214] in Africa, supporting, alongside fossil, archaeological and genomic evidence, the widely accepted African origin of modern humans [54,215]. The out-of-Africa dispersal provides a particularly important case in which molecular-clock estimates can be evaluated against independent palaeoanthropological and archaeological evidence. In the human mtDNA phylogeny, the major non-African founder haplogroups M and N are nested within the African haplogroup L3, and their coalescence ages provide a lower bound for the emergence and early expansion of the maternal lineages ancestral to present-day non-Africans. Using the aforementioned molecular clock, the age of L3 is likely just above 70 ka [38,54,96], while the main non-African founder lineages M and N fall mostly within the range of 70–60 ka [38,96,214,216]. This suggests an out-of-Africa dispersal after 70 ka, but probably closer to 60–65 ka, followed by a rapid movement across southern Asia [95,96,216].

This chronology is also broadly concordant with fossil and archaeological records. In Island and Mainland Southeast Asia, early modern human evidence includes Lida Ajer in Sumatra, dated to approximately 63–73 ka [217], and Tam Pà Ling in Laos, dated to approximately 67–73 ka [216,218]. In Sahul, the oldest secure evidence from northern Australia falls around 53–60 ka, or as old as 53–65 ka under less conservative readings [219]. A dispersal reaching Sunda and Sahul around 60–65 ka is therefore not an isolated molecular inference, but one that fits reasonably with the available archaeological, fossil and palaeoclimatic evidence, including proposed windows of favourable crossings into Sahul around 59–65 ka [216]. The molecular clock dates align with the independent evidence for early modern human presence in Southeast Asia and Australia. The main strength of the mtDNA estimate is not that it provides an exact date for the exit from Africa, but that it yields a coherent chronological framework. However, recent recombination-based estimates place the shared Neanderthal-admixed ancestry of later non-Africans around 47 ka [220,221]. Reconciling these estimates with evidence for modern humans outside Africa before 50 ka remains an open and actively debated question, potentially involving population structure, early dispersals with limited genetic continuity, or methodological differences among the approaches.

The settlement of Remote Oceania provides one of the clearest examples of concordance between molecular-clock estimates and an independently established archaeological chronology. Unlike many older demographic events, the colonisation of Remote Oceania was relatively recent, geographically structured, and well dated by radiocarbon evidence associated with the Lapita expansion and subsequent Polynesian dispersals. It therefore offers a particularly useful test case for founder-age methodology: estimates derived from mtDNA diversity accumulated after settlement should broadly reproduce the independently documented chronology.

Founder-age estimates based on mtDNA haplogroup B4a1a1 [222], including the Polynesian motif, closely match this archaeological sequence: the overall founder age for Remote Oceania was estimated at approximately 3.2 ka [2.7–3.7 ka], in close agreement with the radiocarbon evidence for the initial settlement of Vanuatu and the broader early Remote Oceanian expansion [149,223]. Crucially, these genetic dates were not obtained by imposing the archaeological chronology as a calibration but were estimated from mtDNA diversity accumulated after migration into each island region, using the complete-mitogenome clock [149]. This concordance therefore provides external validation of the founder-age approach and supports the performance of the time-dependent mtDNA clock over recent Holocene timescales, while also justifying its application to more complex and less directly dated regions.

Founder-age analyses using the same time-dependent mtDNA clock have also produced estimates that are broadly concordant with independent demographic and archaeogenetic models in other regions. In Africa, founder ages for maternal lineages entering southern Africa identified a major signal around 2 ka, in agreement with the expected timeframe of the Bantu expansions [224]. In Europe, the inferred arrival times of several haplogroup JT clades showed a close correspondence with their first detection in the aDNA record [225]. Similarly, in Island Southeast Asia, clades displaying a clear phylogeographic signal compatible with the “out-of-Taiwan” model yielded migration estimates around 4 ka [148,150]. Although the latter two cases are not as direct a validation as the Remote Oceanian comparison, because they depend partly on the completeness of the aDNA record or on broader interpretative models, they nevertheless show that founder-age estimates can produce historically coherent results across different regional contexts.

A particularly informative European case concerns the Y chromosome, the paternal counterpart to mtDNA, and illustrates the importance of independent validation for molecular-clock interpretations. Earlier studies of European Y-chromosome variation often interpreted major paternal lineages as reflecting either postglacial recolonisation [226] or Neolithic expansions [227] associated with the spread of farming. Batini et al. (2015), using high-coverage resequencing of 3.7 Mb of the male-specific Y chromosome in 334 males, instead detected a much more recent continent-wide expansion of European patrilineages [228]. Their dating indicated recent coalescent times for the major lineages I1, R1a and R1b, and inferred a demographic expansion beginning approximately 2.1–4.2 ka. The signal subsequently proved highly concordant with aDNA evidence for a major male-mediated expansion from the Pontic-Caspian steppe during the Late Neolithic/Early Bronze Age, associated with the spread of steppe ancestry and the rapid rise of Y-chromosome lineages such as R1a and R1b across much of Europe [229,230,231]. This case therefore provides an instructive example in which molecular-clock estimates were not simply validated by matching a well-defined preconceived archaeological model but gained plausibility through later independent evidence from ancient genomes.

### 6.3. aDNA as Direct Evidence of Age Underestimation

Ancient DNA can also be used not as a calibration point, but as an external check on the plausibility of molecular-clock estimates. If an ancient sample is securely dated and can be confidently assigned to a given clade, then the origin of that clade must predate the age of the sample. Therefore, a molecular-clock estimate that places the clade younger than the ancient sample provides clear evidence that the age has been underestimated, or alternatively that there is an error in sample dating, phylogenetic assignment, or tree reconstruction. This type of comparison is particularly useful because it provides a direct lower bound on clade age that is independent of the molecular-clock model used to generate the estimate [232,233,234].

A particularly direct example of external evaluation using aDNA comes from Barley stripe mosaic virus, a plant RNA virus for which an approximately 750-year-old genome was recovered from archaeological barley at Qasr Ibrim [235]. Molecular-clock analyses based only on modern viral sequences within the same study estimated the crown age of sampled Barley stripe mosaic virus diversity at approximately 50 years and its divergence from its closest known relative around 150 years ago, estimates that were younger than the archaeological genome itself. The ancient sequence therefore provided an unequivocal minimum-age constraint demonstrating that the modern-only analysis had substantially underestimated the temporal depth of the lineage. When the archaeological genome was incorporated as a dated tip, the estimated evolutionary rate decreased and the inferred history of the virus was extended considerably, with its origin most likely placed around 2 ka, although with broad uncertainty.

### 6.4. Humans and Mycobacteria

One particularly informative case that generated discussion about circularity is the molecular clock for *Mycobacterium tuberculosis*, the causative agent of tuberculosis. Comas et al. (2013) [236] analysed whole-genome diversity from the *M. tuberculosis* complex and argued that the pathogen originated in Africa, accompanied anatomically modern humans during the out-of-Africa dispersal, and later expanded with increasing human population density during the Neolithic. However, the absolute dating of this scenario was not based on an independent pathogen calibration. The authors explicitly stated that short-term mutation rates from experimental or epidemiological data could not safely be extrapolated to the deep timescale under consideration, and that tip-to-date analysis showed no significant temporal signal. They therefore calibrated the pathogen phylogeny using key dates from human evolution, including the coalescence of human mtDNA haplogroup L3 at 70 ± 10 ka [54]. The model calibrated on L3 produced dates that appeared to match human dispersal events, including a first split of *M. tuberculosis* lineage 1 around 67 ka and later Eurasian splits broadly compatible with archaeological evidence for modern humans outside Africa. This was interpreted as support for long-term host–pathogen co-divergence, but the argument is largely circular: a human demographic timescale was used to calibrate the bacterial tree, and the resulting bacterial dates were then taken as evidence for concordance with human demographic history. The non-temporal evidence in the study, including African basal lineages, broad phylogeographic similarity between *M. tuberculosis* and human mtDNA, and the statistical association between pathogen lineages and human mtDNA haplogroups, provides independent support for some level of host–pathogen association, but the absolute age of ~70 ka was not independently estimated from the pathogen data. Subsequent work has reinforced this caution. Ancient *M. tuberculosis* genomes [237,238], which provide direct temporal calibration points absent from the original analysis, have generally supported much younger dates for the most recent common ancestor of extant *M. tuberculosis* complex diversity, often in the Neolithic or later. Thus, this *M. tuberculosis* case is best presented not as a straightforward validation of a deep molecular clock, but as a cautionary example. The possibility of a very strong time-dependent effect on the evolutionary rates of *M. tuberculosis* also cannot be excluded.

The animal-adapted member of the complex, *Mycobacterium bovis*, further illustrates the difficulty of distinguishing the age of extant sampled diversity from the deeper history of a disease process. Estimates for the emergence of *M. bovis* have varied substantially depending on the data and calibration strategy. Earlier work placed the coalescence of *M. bovis* within a broadly Neolithic timeframe, around or after the spread of animal domestication, with estimates of at least ~6 ka [239] and a broader interpretative context linked to cattle domestication beginning in the Near East around 10 ka. This timescale is compatible with phylogeographic analyses of African *M. bovis*, where diversity is geographically structured and deepest in Eastern and Northern Africa, broadly matching the archaeological sequence for the introduction of domesticates and pastoralism across the continent [240]. More recent genome-based analyses have inferred a much younger age, around 0.7–3.6 ka [241], for the most recent common ancestor of sampled/extant *M. bovis* lineages. However, this younger estimate is difficult to interpret as the first spread of bovine tuberculosis in Africa, because it would require either substantial lineage replacement, strong bottlenecks, or loss of earlier diversity to explain the observed regional structure [242]. Thus, as with *M. tuberculosis,* the molecular dates for extant *M. bovis* lineages remain difficult to interpret.

## 7. Conclusions

Molecular clocks remain among the most powerful tools for transforming genetic variation into historical inference. They have allowed researchers to estimate the timing of species divergences, population expansions, pathogen emergence, migration events and major episodes in the history of life. However, the apparent simplicity of the metaphor should not obscure the complexity of the process. Genetic change does not accumulate as a universal stopwatch. Mutation and substitution rates vary across genomes, taxa, lineages, environments and timescales, and the rate inferred from one dataset or temporal depth cannot automatically be transferred to another. For this reason, molecular-clock analyses should begin not with the assumption that a clock exists, but with the evaluation of whether the data contain sufficient temporal signal and whether the chosen clock model is biologically and statistically appropriate.

The central challenge is calibration. Branch lengths alone provide relative genetic divergence; only calibration converts those distances into absolute time. Fossils, dated samples, geological events, founder events, pedigrees and directly measured mutation rates can all provide temporal information, but each carries its own assumptions and biases. Secondary calibrations and externally estimated rates can be useful when no direct calibration is available, but they should be treated as priors with uncertainty rather than as independent empirical anchors. Similarly, agreement between molecular dates and external historical events can be informative, but only when the same events have not been used to generate the dates in the first place. The strongest molecular-clock studies are therefore those that combine careful data curation, explicit model comparison, transparent calibration choices, sensitivity analyses and independent validation where possible.

Molecular clocks should not be viewed as mechanisms that automatically reveal evolutionary time, but as inferential frameworks whose strength depends on how carefully their assumptions are tested and how cautiously their results are interpreted. Looking ahead, developments in artificial intelligence and machine learning may become increasingly useful for molecular-clock analyses by accelerating model selection, detecting rate heterogeneity, and identifying datasets with weak temporal signal or violations of clock assumptions. Recent work has shown that machine learning can match or approach ML performance for some phylogenetic tasks and can speed up model selection, suggesting a role for artificial intelligence as a diagnostic and pre-analysis tool rather than as a substitute for mechanistic clock models [243,244,245].

## Data Availability

No new data were created or analyzed in this study. Data sharing is not applicable to this article.

## Abbreviations

The following abbreviations are used in this manuscript:

aDNA	Ancient DNA
BETS	Bayesian evaluation of temporal signal
BI	Bayesian Inference
FBD	Fossilized birth–death
DRT	date-randomization tests
ML	Maximum likelihood
MP	Maximum Parsimony
mtDNA	Mitochondrial DNA
